# Thwarted Belongingness Hindered Successful Aging in Chinese Older Adults: Roles of Positive Mental Health and Meaning in Life

**DOI:** 10.3389/fpsyg.2022.839125

**Published:** 2022-02-24

**Authors:** Yongju Yu

**Affiliations:** Department of Social Work, School of International Law and Sociology, Sichuan International Studies University, Chongqing, China

**Keywords:** thwarted belongingness, successful aging, meaning in life, positive mental health, older adults

## Abstract

Aging of population has brought great challenges to many regions throughout the world. It has been demonstrated that interpersonal relationship is closely related to the experiences of aging for older adults. However, it still remains unknown how and under what conditions thwarted belongingness links to successful aging. This study examined the relationship between thwarted belongingness and successful aging and tested the mediating role of positive mental health and the moderating role of meaning in life. Community-dwelling older adults (*n* = 339) aged 60–75 years recruited in Chongqing, China completed self-measures of thwarted belongingness, successful aging, meaning in life, and positive mental health. Correlation analyses showed that successful aging was associated with less thwarted belongingness, better positive mental health, and higher levels of meaning in life. Positive mental health was found to totally mediate the negative effect of thwarted belongingness on successful aging. Moderated mediation analyses further revealed that two components of meaning in life (present of meaning and search for meaning) attenuated the indirect effect of thwarted belongingness on successful aging *via* positive mental health. This study highlights the protective roles of positive mental health and meaning in life and addressed cultural aspects in the process of successful aging among Chinese older adults.

## Introduction

At present, the population aging is increasing with an irresistible trend, which is a huge challenge for many regions. China is one of the countries with the fastest growing population aging in the world (Huang, 2020; Luo et al., 2020). According to the data of the Chinese seventh national census (National Bureau of Statistics of China, 2021), China’s population aged 60 and over was 264.02 million, accounting for 18.70% of the population. Among them, the population aged 65 and over was 190.64 million, accounting for 13.50%. The acceleration in the population aging would produce a series of problems, such as the decline of labor supply, the weakening of consumer demand, as well as the decline of household savings and economic growth (Dollar et al., 2020; Huang, 2020). Meanwhile, it can increase the pressure on social security and public services (Wang and Zhou, 2020). “Better with age,” that is, successful aging, has become a hot issue in the field of aging research. Successful aging is defined as an individual’s perception of favorable adaptation to the cumulative physiological and functional alterations associated with the passage of time, while experiencing spiritual correctness and a sense of meaning and purpose in life (Flood, 2002; Troutman et al., 2011b).

### Thwarted Belongingness, Positive Mental Health, and Successful Aging

According to the MacArthur’s theory (Rowe and Kahn, 2015; Jang and Kim, 2021), successful aging encompasses three principal components: low risk of disease and disease-related disability, maintenance of high mental and physical function, and continued engagement with life, which includes relations with others and productive activity. It has been demonstrated that interpersonal relationships are closely related to the experiences of aging for older adults (Shiovitz-Ezra and Litwin, 2012; Ye and Zhang, 2021). Due to the changes of social status and functional limitations, older people are more vulnerable to social isolation and relationship breakdown than others (Stoeckel and Litwin, 2016). It is of great importance to pay attention to older adults’ interpersonal needs, which reflect their sense of self-worth and belongingness. According to the finding by Van Orden et al. (2010), interpersonal needs consist of two related but independent components: thwarted belongingness and perceived burdensomeness. Thwarted belongingness is a lack of social relationships, which is accompanied by loneliness. Perceived burdensomeness is the thought that one is a burden upon one’s loved ones and is accompanied by self-hatred. Insufficient interpersonal needs may make individuals feel pain due to lack of connection with others, thereby resulting in non-adaptive behaviors (Kwon et al., 2020). Widely recognized by researchers, a high degree of frustration of interpersonal needs is considered to be an important indicator for suicide ideation (Park and Kim, 2019; Kyron et al., 2021).

However, few studies to date have directly examined the relationship between interpersonal needs and successful aging. Nevertheless, the link between interpersonal needs and the living quality of aging people was also evidenced. A recent study on community-dwelling older adults showed that interpersonal needs had a great impact on attitude toward aging (Jang and Kim, 2021). A sense of belongingness helps individuals to express their identity, promote emotional well-being in their relationships, and enhance physical and mental health (Shields, 2008). On the contrary, low senses of belonging and perceived psychological or emotional burdens are considered to be risk factors of mortality in older adults due to psychological problems such as loneliness, hopelessness, and depression (Van Orden et al., 2012). Previous studies found that older adults scored significantly higher on thwarted belongingness than on perceived burdensomeness assessed by the Likert scale (Eades et al., 2019; Kinory et al., 2020). This indicated that thwarted belongingness is a more common psychological experience than perceived burdensomeness for most older adults. Accordingly, only thwarted belongingness was examined in this study. We speculate that thwarted belongingness would influence older adults’ positive mental health, and further affect quality of aging (i.e., successful aging). In this study, the impact of positive feelings and positive functioning of older adults on successful aging was examined. Therefore, the term of “positive mental health” proposed by Keyes (2002) was used.

### The Moderating Role of Meaning in Life

As a cognitive coping resource, meaning in life is an important protective factor when people face major setbacks (Zhong et al., 2019; Yu et al., 2021). Meaning in life refers to an innate drive to find meaning and significance in individuals’ lives (Steger et al., 2006). Ego integrity is the core issue that a person faces in his/her late life (Erikson, 1982). Bueno-Pacheco et al. (2021) demonstrated that trying to find meaning and reconciling life events can facilitate the achievement of ego integrity. Finding and maintaining meaning and purpose in one’s life (i.e., meaning in life) has been demonstrated to play an important role in the relationship between stressful events and mental health. Researchers revealed that interventions aimed at increasing meaning and purpose in life can improve individuals’ health and well-being in older adults (Gellis et al., 2020). An empirical study on 588 older adults showed that higher levels of meaning and goal could buffer the impacts of interpersonal stressful events on depressive symptoms and life satisfaction (Lee et al., 2022). Conversely, reduced meaning in life was found to be a crucial predictor to the feeling of loneliness (Macià et al., 2021). Accordingly, we hypothesized that meaning in life would moderate the relationship between thwarted belongingness, positive mental health, and successful aging in older adults, which will be tested in the current study.

To date, the most widely used self-report instrument to assess the meaning of life is the Meaning in Life Questionnaire (MLQ) developed by Steger et al. (2006). It has two factors: presence of meaning (MLQ_P) and search for meaning (MLQ_S). Previous studies hold generally consistent viewpoints on the effect of MLQ_P and addressed its importance in facilitating mental health, promoting life functioning, and reducing emotional distresses (Steger et al., 2009; Yang et al., 2019). By contrast, the impact of MLQ_S on mental health outcomes is controversial. Some researchers asserted that search for meaning is painful, which is usually positively correlated with depression, anxiety, depression, and negative self-concept (Steger et al., 2006). Search for meaning may lead to increased depression and anxiety as well as a stronger sense of loss in the late life (Davis et al., 2000). However, several studies in Eastern countries found that the relationship between search for meaning and mental health outcomes was non-significant or even positive (Liu and Gan, 2010; Jin et al., 2016; Yang et al., 2019).

This phenomenon may be due to culture difference. Steger et al. (2008) found people in Eastern countries usually hold a dialectical way of thought, and regard the continuous process of searching for meaning as essential to acquire desired outcomes. Therefore, in our study, the moderating roles of MLQ_P and MLQ_S were tested, respectively. We speculate that both of these two components act as beneficial factors in facilitating older adults’ mental health outcomes in the Chinese context.

### The Current Study

**FIGURE 1:**
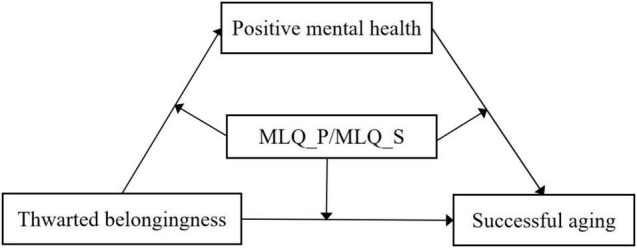
The moderation mediation model showing the relationships among thwarted belongingness, positive mental health, meaning in life, and successful aging. MLQ_P, presence of meaning; MLQ_S, search for meaning.

Due to gaps in these existing literature (i.e., little research with older adults about thwarted belongingness, positive mental health, meaning in life, and successful aging), the current study sought to clarify how and under what conditions thwarted belongingness links to successful aging, and also test the roles of positive mental health and meaning in life among Chinese older adults. Based on the MacArthur’s theory and existing evidence, a framework was developed in the current study (see Figure 1). The specific hypotheses were as follows:

**Hypothesis 1:** Successful aging would go along with less thwarted belongingness, better positive mental health, and more meaning in life in Chinese older adults.
**Hypothesis 2:** Positive mental health would mediate the relationship between thwarted belongingness and successful aging.
**Hypothesis 3:** Presence of meaning (MLQ_P) and search for meaning (MLQ_S) would serve as beneficial factors and moderate the pattern of relationships between thwarted belongingness, positive mental health, and successful aging.

## Materials and Methods

### Participants and Procedures

The investigation was conducted from February to October, 2021. Authorization for the data collection was obtained from the Ethics Committee of Sichuan International Studies University. The inclusion criteria of participants in this study were (1) aged between 60 and 75; (2) living in communities rather than nursing institutions; (3) no history of serious psychiatric or neurological illness; and (4) no evidence of substance abuse or dependence in the past 3 months. Through flyers and advertisements, a total of 362 older adults were recruited from 4 communities in Chongqing, China. Before investigation, the inclusion criteria and the study goals were explained to all participants. After signing the written informed consent, all participants completed self-reported measures of background information, thwarted belongingness, successful aging, meaning in life, and positive mental health. For the participants who could not read or understand questionnaires independently, the investigators read questions one by one and helped them fill in the questionnaires. After excluding 23 incompletely filled questionnaires, a total of 339 valid questionnaires were obtained. The final samples consisted of 133 males and 206 females. The mean age of valid participants was 65.32 (SD = 3.43). Table 1 provides the demographic characteristics of older adults in the current study.

**TABLE 1:** Demographic characteristics of participants.

Variables	*N* (%)	Variables	*N* (%)
Gender		Education level	
Male	133 (39.2)	Less than junior middle school	63 (18.6)
Female	206 (60.8)	Junior middle school	142 (41.9)
Economic status		High school	103 (30.4)
Poor	28 (8.3)	College/bachelor degree or above	31 (9.1)
A little poor	160 (47.2)	Marital status	
A little rich	134 (39.5)	Single	15 (4.5)
Rich	17 (5.0)	Married	283 (83.3)
		Window/divorced	41 (12.2)

### Study Measures

#### Thwarted Belongingness

The thwarted belongingness subscale of Interpersonal Needs Questionnaire (INQ; Van Orden et al., 2012; Lai and Boag, 2021) was adopted to assess what extent the respondent feels connected to others. Nine items are presented on a 7-point scale, ranging from 1 (not at all true for me) to 7 (very true for me). For example, “these days, other people care about me.” It has been demonstrated adequate reliability and validity in the Chinese samples (Lai and Boag, 2021; Wang et al., 2021). The Cronbach’s alpha value for the thwarted belongingness subscale in our sample was 0.71.

#### Successful Aging

The 20-item Successful Aging Inventory (SAI; Troutman et al., 2011b; Cheng, 2014) was used to measure older adults’ level of successful aging. For example, “I feel interest in/concern for the next generation.” Participants were asked to rate the items on a 5-point Likert-type scale from 0 (never) to 4 (always). This scale has been demonstrated adequate reliability and validity in the Chinese sample (Cheng, 2014). The Cronbach’s alpha value for the total scale was 0.89 in our sample.

#### Meaning in Life

The 10-item MLQ was developed (Steger et al., 2006) to measure individuals’ perceived meaning in life. It consists of two 5-item factors: presence of meaning (labeled MLQ_P, e.g., “I have a good sense of what makes my life meaningful”) and search for meaning (labeled MLQ_S, e.g., “I am seeking a purpose or mission for my life”). Participants were asked to rate the items on a 7-point Likert-type scale from 1 (absolutely untrue) to 7 (absolutely true). The MLQ has been demonstrated adequate reliability and validity in the Chinese samples (Wang and Dai, 2008). In this study, the Cronbach’s alpha values for the Presence subscale, the Search subscale, and the total scale were 0.74, 0.81, and 0.87, respectively.

#### Mental Health

The 14-item Mental Health Continuum-Short Form (MHC-SF; Keyes, 2002; Yin and He, 2012) integrates three components (emotional well-being, psychological well-being, and social well-being) as indicators to evaluate positive mental health. Participants were asked to rate how often they felt a certain way during the past month, on a 6-point scale from 0 (never) to 5 (every day). For example, “I am good at managing the responsibilities of daily life.” The higher the score, the better the individual’s mental health is. This scale has been proved to have good reliability, acceptability, and validity (Yin and He, 2012). Total score of MHC-SF was used in the current study. The Cronbach’s alpha value for the total scale of MHC-SF was 0.92 in this sample.

### Data Analyses

According to Podsakoff et al.’s (2003) suggestions, Harman’s one-factor test was adopted to test the potential common method biases for all research items. Nine distinct factors with eigenvalue greater than 1 were obtained, with the largest factor accounting for 34.94% of the variance (<40%, the threshold level). Therefore, the common method variance was limited in the current study. Descriptive analyses were used to describe the values of study variables. Independent sample *t*-test and one-way ANOVA were performed to test the effects of gender and marital status on successful aging. Pearson correlation analyses were conducted to explore the associations of study variables. Hierarchical linear regression analyses were used to examine whether positive mental health mediated the link of thwarted belongingness and successful aging in older adults. PROCESS macro for SPSS with bootstrapping (Hayes, 2017) was performed to examine the moderating roles of MLQ_P and MLQ_P in the relationship between thwarted belongingness, positive mental health, and successful aging. Considering the significant correlations with successful aging, education level and physical health were controlled as covariates in the statistical analyses. All continuous variables were centered before testing the hypothesized moderated mediation model. SPSS 26.0 was used for data analyses in the current study.

## Results

### The Characteristics and Correlations of Study Variables

Means, standard deviations, and possible ranges were calculated for main study variables in the current study. Independent sample *t*-test was performed and the results showed that no gender difference was found in successful aging between men and women (52.73 ± 12.15 vs 51.78 ± 12.06, *t* = 0.70, *p* = 0.482 > 0.05). One-way ANOVA showed that the effect of marital status on successful aging did not reach statistical significance [*F*_(2,336)_ = 1.354, *p* = 0.260 > 0.05]. A series of Pearson correlation analyses were conducted to explore possible associations among main study variables. Results in Table 2 showed that there was no significant relationship between age, economic status, and successful aging (*p*s > 0.05). However, successful aging was found to be related to higher levels of education (*r* = 0.26, *p* < 0.001) and better physical health (*r* = 0.25, *p* < 0.001). As predicted, successful aging was negatively related to thwarted belongingness (*r* = −0.52, *p* < 0.001) and positively related to MLQ_P, MLQ_S, and positive mental health (*r* = 0.57–0.83, *ps* < 0.001). Accordingly, the initial Hypothesis 1 was well supported.

**TABLE 2:** Descriptive statistics and correlations between variables.

	Mean ± SD	TB	MLQ_P	MLQ_S	Positive mental health	Successful aging
TB	30.64 ± 7.70	1				
MLQ_P	23.29 ± 4.47	−0.52***	1			
MLQ_S	24.91 ± 5.34	−0.39***	0.66***	1		
Positive mental health	46.36 ± 12.72	−0.57***	0.58***	0.61***	1	
Successful aging	52.15 ± 12.09	−0.52***	0.57***	0.65***	0.83***	1
Age	65.32 ± 3.43	–0.01	–0.10	−0.13*	–0.04	–0.01
Economic status		−0.12*	0.15**	0.05	0.09	0.06
Education level		−0.21***	0.27***	0.21***	0.22***	0.26***
Physical health		−0.22***	0.22***	0.14*	0.28***	0.25***

n = 339. TB, thwarted belongingness; MLQ_P, presence of meaning; MLQ_S, search for meaning. *p < 0.05; **p < 0.01; ***p < 0.001.

### Testing for the Mediation Model With Positive Mental Health as the Mediator

Hierarchical linear regression analyses were used to test whether positive mental health served as a mediator in the link of thwarted belongingness and successful aging. Model 1 regressed positive mental health on thwarted belongingness after controlling for education level and physical health. Model 2 regressed successful aging on thwarted belongingness with education level and physical health as covariates. The results indicated that thwarted belongingness had a significant negative influence on older adults’ positive mental health (β = −0.52, *t* = −11.43, *p* < 0.001) and successful aging (β = −0.46, *t* = −9.75, *p* < 0.001). Model 3 regressed successful aging on thwarted belongingness and positive mental health after controlling for education level and physical health. Our results revealed that thwarted belongingness did not significantly predict successful aging (β = −0.05, *t* = −1.46, *p* = 0.146 > 0.05) after positive mental health was entered into the regression model, while positive mental health exhibited a positive impact on successful aging (β = 0.78, *t* = 20.98, *p* < 0.001). The mediation model can explain 70.2% of successful aging variance. Accordingly, positive mental health totally mediated the effect of thwarted belongingness on successful aging. Therefore, our initial Hypothesis 2 was supported.

### Testing for the Moderated Mediation Model With MLQ_P as the Mediator

Hayes’s (2017) PROCESS macro with Model 59 was applied to test whether presence of life (MLQ_P) could moderate the relationships between thwarted belongingness, positive mental health, and successful aging. As shown in Table 3, after controlling for education level and physical health, thwarted belongingness negatively predicted positive mental health (*b* = −0.67, *t* = −8.20, *p* < 0.001), and this effect was moderated by MLQ_P (*b* = 0.04, *t* = 3.39, *p* < 0.001). Consistent with the results of the above mediation model, thwarted belongingness could not directly predict successful aging (*b* = −0.11, *t* = −1.65, *p* = 0.099 > 0.05), while positive mental health positively predicted successful aging (*b* = 0.69, *t* = 19.09, *p* < 0.001). In addition, the link between positive mental health and successful aging was moderated by MLQ_P (*b* = 0.02, *t* = 2.14, *p* = 0.001 < 0.05). Therefore, MLQ_P served as a mediator in the relationship between thwarted belongingness and positive mental health (the first stage of the mediating effect) as well as the relationship between positive mental health and successful aging (the second stage of the mediating effect).

**TABLE 3:** Testing the moderated mediation effect (MLQ_P as a mediator).

Dependent variables	Predictors	*b*	SE	*t*	*R* ^2^	*F*
Positive mental health					0.467	58.391***
	Education level	0.28	0.59	0.48		
	Physical health	1.74	0.58	3.02**		
	TB	–0.67	0.08	−8.20***		
	MLQ_P	0.95	0.14	6.92***		
	MLQ_P × TB	0.04	0.01	3.39***		
Successful aging					0.716	119.315***
	Education level	0.78	0.41	1.89		
	Physical health	0.04	0.41	0.10		
	TB	–0.11	0.06	–1.65		
	Positive mental health	0.69	0.04	19.09***		
	MLQ_P	0.31	0.10	2.99**		
	MLQ_P × TB	0.03	0.12	2.85**		
	MLQ_P × positive mental health	0.02	0.01	2.14*		
Conditional indirect effect analysis		MLQ_P value	Effect	Boot SE	BootLLCI	BootULCI
		18.82	−0.52***	0.08	–0.71	–0.38
		23.29	−0.46***	0.07	–0.60	–0.33
		27.76	−0.37***	0.09	–0.54	–0.19

The b values are unstandardized coefficients. TB, thwarted belongingness; MLQ_P, presence of meaning. *p < 0.05; **p < 0.01; ***p < 0.001.

Furthermore, the conditional indirect effect analysis showed that the indirect effect of thwarted belongingness on successful aging *via* positive mental health was moderated by MLQ_P (Table 3). For older adults with low levels of MLQ_P, thwarted belongingness exhibited a negative impact on successful aging through decreased positive mental health (*b* = −0.52, *p* < 0.001). For older adults with high levels of MLQ_P, this indirect effect remained significant, but to a weaker degree (*b* = −0.37, *p* < 0.001).

**FIGURE 2:**
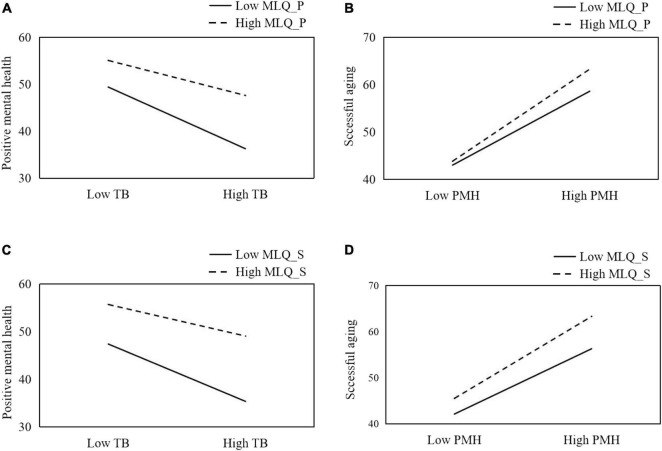
MLQ_P and MLQ_S moderated the pattern of relationships between thwarted belongingness, mental health, and successful aging. **(A)** The moderating effect of MLQ_P on the link between thwarted belongingness and mental health; **(B)** the moderating effect of MLQ_P on the link between mental health and successful aging; **(C)** the moderating effect of MLQ_S on the link between thwarted belongingness and mental health; **(D)** the moderating effect of MLQ_S on the link between mental health and successful aging. TB, thwarted belongingness; MLQ_P, presence of meaning; MLQ_S, search for meaning; PMH, positive mental health.

According to Holmbeck’s (2002) guidelines, we examined simple slopes at 1 SD above and below the mean MLQ_P level to test the significant effect of the MLQ_P × thwarted belongingness interaction on positive mental health. As shown in Figure 2A, at low levels of MLQ_P (<18.82), thwarted belongingness exhibited a significant and negative impact on positive mental health (β = −0.51, *t* = −7.74, *p* < 0.001). At high levels of MLQ_P (>27.76), thwarted belongingness could also significantly predict positive mental health (β = −0.29, *t* = −5.60, *p* < 0.001). These results indicated that thwarted belongingness had a stronger impact on positive mental health for older adults having lower levels of present of meaning, compared with those reporting higher levels of present of meaning. To illustrate the moderating effect of MLQ_P in the relationship between positive mental health and successful aging, another simple slope test was performed. As shown in Figure 2B, at low levels of MLQ_P, positive mental health exhibited a significant and positive impact on successful aging (β = 0.64, *t* = 12.13, *p* < 0.001). At high levels of MLQ_P, positive mental health could also significantly successful aging (β = 0.81, *t* = 14.36, *p* < 0.001). The results revealed that the effect of positive mental health on successful aging was weaker for older adults with lower levels of present of meaning, as compared to those who had higher levels of present of meaning.

### Testing for the Moderated Mediation Model With MLQ_S as the Mediator

The same procedure was performed to test the moderating role of search for meaning (MLQ_S). As shown in Table 4, thwarted belongingness could negatively predict positive mental health (*b* = −0.61, *t* = −8.80, *p* < 0.001), and this effect was moderated by MLQ_S (*b* = 0.03, *t* = 3.04, *p* = 0.003 < 0.01). Thwarted belongingness could not directly predict successful aging (*b* = −0.08, *t* = −1.46, *p* = 0.146 < 0.001). Successful aging could be positively predicted by positive mental health (*b* = 0.63, *t* = 15.97, *p* < 0.001) and this effect was moderated by MLQ_S (*b* = 0.013, *t* = 1.23, *p* = 0.034 < 0.05). These results indicated that MLQ_S moderated the relationship between thwarted belongingness and positive mental health (the first stage of the mediating effect) and it also moderated the relationship between positive mental health and successful aging (the second stage of the mediating effect).

**TABLE 4:** Testing the moderated mediation effect (MLQ_S as a mediator).

Dependent variable	Predictor	*b*	SE	*t*	*R* ^2^	*F*
Mental health					0.53	76.03***
	Education level	0.31	0.55	0.56		
	Physical health	2.07	0.54	3.81***		
	TB	–0.61	0.07	−8.80***		
	MLQ_S	1.03	0.10	10.49***		
	MLQ_S × TB	0.03	0.01	3.04**		
Successful aging					0.74	131.22***
	Education level	0.86	0.39	2.18*		
	Physical health	0.10	0.40	0.25		
	TB	–0.08	0.06	–1.46		
	Mental health	0.63	0.04	15.97***		
	MLQ_S	0.49	0.08	6.01***		
	MLQ_S × TB	0.013	0.011	1.23		
	MLQ_S × mental health	0.013	0.006	2.13*		
Conditional indirect effect analysis		MLQ_S value	Effect	Boot SE	BootLLCI	BootULCI
		19.57	−0.44***	0.07	–0.58	–0.31
		24.91	−0.38***	0.05	–0.48	–0.28
		30.26	−0.30***	0.07	–0.44	–0.17

The b values are unstandardized coefficients. TB, thwarted belongingness; MLQ_S, search for meaning. *p < 0.05; **p < 0.01; ***p < 0.001.

Furthermore, the conditional indirect effect analysis showed that the indirect effect of thwarted belongingness on successful aging *via* positive mental health was moderated by MLQ_S (Table 4). For older adults with low levels of MLQ_S, thwarted belongingness exhibited a negative impact on successful aging through decreased positive mental health (*b* = −0.44, *p* < 0.001), although this indirect effect was still significant but became weaker (*b* = −0.30, *p* < 0.001) for older adults with high levels of MLQ_P.

The simple slope tests shown in Figure 2C revealed that when low MLQ_S was reported by older adults, the linkage of thwarted belongingness and positive mental health was significant (β = −0.46, *t* = −8.75, *p* < 0.001). When MLQ_S was high, thwarted belongingness could also predict positive mental health but became weaker (β = −0.25, *t* = −4.30, *p* < 0.001). As illustrated in Figure 2D, the relationship between positive mental health and successful aging was weaker (β = 0.59, *t* = 11.44, *p* < 0.001) for participants reporting lower levels of MLQ_S, compared to those reporting higher levels of MLQ_S (β = 0.75, *t* = 12.79, *p* < 0.001).

Together, the above results substantiated our Hypothesis 3 that MLQ_P and MLQ_S serve as beneficial factors and moderate the pattern of relationships between thwarted belongingness, positive mental health, and successful aging.

## Discussion

Previous studies have indicated the close relationship between interpersonal relations and successful aging (Van Orden et al., 2012; Jang and Kim, 2021). Based on the MacArthur’s theory, the current study extends the previous finding by identifying the mechanisms connecting thwarted interpersonal needs to successful aging. Our study generally supported initial three hypotheses. Specifically, the results revealed that successful aging was related to less thwarted belongingness, better positive mental health, and more meaning in life in Chinese older adults. Positive mental health can fully mediate the relationship between thwarted belongingness and successful aging. The indirect effect of thwarted belongingness on successful aging through positive mental health was moderated by two factors of meaning in life (i.e., presence of meaning and search for meaning).

Older adults in the current study exhibited higher levels of thwarted belongingness and poorer quality of successful aging. Specifically, single-sample *t*-tests showed that the mean total score of thwarted belongingness reported by older adults in our study (30.64 ± 7.07) was significantly higher than the findings reported by Eades et al. (2019) (19.87 ± 12.04, *t* = 25.78, *p* < 0.001, Cohen’s *d* = 1.06) and Shim et al. (2021) (22.76 ± 9.29, *t* = 18.85, *p* < 0.001, Cohen’s *d* = 0.92). The mean total score of successful aging in our sample was significantly lower than the results reported by Troutman et al. (2011a) (52.15 ± 12.09 vs 63.48 ± 11.23, *t* = −17.25, *p* < 0.001, Cohen’s *d* = −0.97). These results indicated that older adults in the current study generally had frustrated interpersonal needs and poor quality of successful aging. Our study revealed that most of socio-economic variables (age, gender, economic status, and marital status) had no significant association with successful aging, consistent with the previous finding (Kim and Park, 2016). Education level and physical health were found to have positive associations with successful aging in our study. This is understandable since education acts as a proxy for other factors such as social resources, coping style, etc. (Bluth et al., 2020; Yu and Xiao, 2021). The higher the education level, the better the positive mental health and quality of life for older adults will be. Meanwhile, good physical function and health conditions help older adults to expand activity space and enrich their social life, thereby archiving successful aging in later life (Kim and Park, 2016). Conversely, severe physical diseases or function limitations can affect individuals’ social activities and hinder the process of successful aging (Fingerman et al., 2021).

In line with previous studies (Jang and Kim, 2021; Ye and Zhang, 2021), our results of correlation analyses clearly supported the Hypothesis 1 that successful aging is closely attached to less thwarted belongingness, better mental health, and more meaning in life in Chinese older adults. Regarding the relationship between thwarted belongingness and successful aging, our finding lends support to the MacArthur model (Rowe and Kahn, 2015), which addressed that successful aging is closely associated with maintenance good relations with others. The close relationship between successful aging, positive mental health, and meaning in life is also easily understandable, since meaning in life relates to one’s interpretation of their experiences (Lee et al., 2022), protects individuals against boredom and emptiness (Melton and Schulenberg, 2007), and improves psychological well-being and life satisfaction (Zhou and Xu, 2019).

Researchers have emphasized the importance of relationships for quality of life in aging by stating that social interaction can promote the exchange of feelings capable of enhancing or mitigating the offer and receipt of assistance related to health maintenance (Soares et al., 2021). Conversely, negative social interaction and interpersonal conflict often have a great impact on older adults’ psychological well-being (Hupkens et al., 2018; Lee et al., 2022). In line with previous studies, mediation analyses in the current study revealed that the effect of thwarted belongingness on successful aging was totally carried by the reduction of positive mental health. Accordingly, positive mental health played an important role in interpreting why and how thwarted belongingness is associated with poorer quality of successful aging. Therefore, initial Hypothesis 2 in our study was well supported.

Moderation analyses showed that meaning in life emerged as a moderator in the relationship between thwarted belongingness and positive mental health. In our study, pursing (searching for meaning) and maintaining meaning in life (presence of meaning) could attenuate the indirect effect of thwarted belongingness on successful aging *via* positive mental health. This is congruent with the previous finding that meaning in life works following the protective-protective model (Cohen et al., 2003; Yang et al., 2019). Specifically, our results demonstrated that presence of meaning buffered the link between thwarted belongingness and positive mental health, therefore the protective-attenuating role of presence of meaning was confirmed. Besides that, presence of meaning strengthened the link between positive mental health and successful aging, indicating the protective-enhancing role of presence of meaning was also revealed. Highly consistent with the role of presence of meaning, searching for meaning also works following the protective-protective model. These results confirmed the existential theory that an increased sense of meaning and purpose in life is often linked to greater stress coping capacity (Frankl, 1984; Macià et al., 2021) and better outcomes on a range of health and well-being (Yang et al., 2019). Therefore, thwarted belongingness exhibited less adverse effects on positive mental health and the protective effect of positive mental health on successful aging was strengthened for older adults with actively finding and maintaining meaning and purpose. Consequently, these results well supported our Hypothesis 3 that presence of meaning and search for meaning serve as beneficial factors and moderate the pattern of relationships between thwarted belongingness, positive mental health, and successful aging.

Previous studies in Western countries (Cohen and Cairns, 2011; Gellis et al., 2020) have asserted that for older persons, an ongoing search for meaning in life is linked to negative outcomes than a perception of existing meaning in life. However, our study underlines the beneficial role of search for meaning in the process of successful aging, which is consistent with the studies conducted in Eastern countries (Steger et al., 2008; Jin et al., 2016; Yang et al., 2019). Therefore, the value systems and cultural aspects should be considered in the process of successful aging (Hyun Cha et al., 2012). For older adults who actively seek goals and meaning in life, they may regard thwarted belongingness as momentary and controllable. They are more inclined to regulate their activities to establish new beliefs and goals rather than immersing themselves in the negative experiences.

### Limitations

Some limitations of this study should be noted. Firstly, due to the cross-sectional design, the causal relationship of main study variables should be further confirmed in future studies. Secondly, older adults with 60–75 years old were recruited in our study. They still have better psychical and mental functions to find new goals and values of life. Therefore, these conclusions in our study can not be applied to people who aged above 75 years old. Meanwhile, the limited age range of older adults in this study prevented us from clarifying the relationship between age and successful aging. Thirdly, the sample of 339 older adults is indeed a small one which may limit the generalization of the results. The findings in the current study need to be further verified in larger samples. Finally, there might be sampling bias in this study. It is hardly accessible to recruit those who have more serious social isolation and thwarted belongingness due to severe physical limitations.

## Conclusion

The present study illustrates how and under what conditions thwarted belongingness exhibits the negative effect on successful aging. First, we demonstrated that thwarted belongingness hindered the process of successful aging by decreased positive mental health. Second, it was found that both presence of life and search for meaning moderate the indirect negative effect of thwarted belongingness on successful aging through positive mental health, with this negative effect being weaker for individuals with higher levels of meaning in life. Accordingly, promoting older adults’ meaning and purpose in life may be an effective avenue to ameliorate the negative experiences of thwarted belongingness and enhance the process of successful aging. These findings have significant implications for the development of successful aging, which may offer useful guidance on professional counseling for Chinese older adults.

## Data Availability Statement

The raw data supporting the conclusions of this article will be made available by the authors, without undue reservation.

## Ethics Statement

The studies involving human participants were reviewed and approved by the Ethics Committee of Sichuan International Studies University. The patients/participants provided their written informed consent to participate in this study.

## Author Contributions

YY designed the study, collected and analyzed the data, and wrote the manuscript independently. The author contributed to the article and approved the submitted version.

## Conflict of Interest

The author declares that the research was conducted in the absence of any commercial or financial relationships that could be construed as a potential conflict of interest.

## Publisher’s Note

All claims expressed in this article are solely those of the authors and do not necessarily represent those of their affiliated organizations, or those of the publisher, the editors and the reviewers. Any product that may be evaluated in this article, or claim that may be made by its manufacturer, is not guaranteed or endorsed by the publisher.

## References

[B1] Bluth K. Park J. Lathren C. (2020). Is parents’ education level associated with adolescent self-compassion? *Explore* 16 225–230. 10.1016/j.explore.2020.02.003 32245709 PMC7654721

[B2] Bueno-Pacheco A. Satorres E. Delhom I. Meléndez J. C. (2021). Ego-integrity and its relationship with sense of coherence, satisfaction, self-efficacy, and depression. *Curr. Psychol.* [Epub online ahead of print]. 10.1007/s12144-021-01978-z

[B3] Cheng Y. L. (2014). *Research of Reliability and Validity of the Chinese Version Successful Aging Inventory and its Application in Quality of Life (in Chinese).* Jinan: Shandong University.

[B4] Cohen J. Cohen P. West S. G. Aiken L. S. (2003). *Applied Multiple Regression/Correlation Analysis for the Behavioral Sciences(3rd ed.).* Hillsdale: Erlbaum.

[B5] Cohen K. Cairns D. (2011). Is searching for meaning in life associated with reduced subjective well-being? Confirmation and possible moderators. *J. Happiness Stud.* 13 313–331. 10.1007/s10902-011-9265-7

[B6] Davis C. G. Wortman C. B. Lehman D. R. Silver R. C. (2000). Searching for meaning in loss: are clinical assumptions correct? *Death Stud.* 24 497–540. 10.1080/07481180050121471 11503666

[B7] Dollar D. Huang Y. Yao Y. (2020). *China 2049: Economic Challenges of a Rising Global Power.* Washington: Brookings Institution Press.

[B8] Eades A. Segal D. L. Coolidge F. L. (2019). Suicide risk factors among older adults: exploring thwarted belongingness and perceived burdensomeness in relation to personality and self-esteem. *Int. J. Aging Hum. Dev.* 88 150–167. 10.1177/0091415018757214 29480062

[B9] Erikson E. H. (1982). *The Life Cycle Completed.* New York, NY: Norton.

[B10] Fingerman K. L. Ng Y. T. Huo M. Birditt K. S. Charles S. T. Zarit S. (2021). Functional limitations, social integration, and daily activities in late life. *J. Gerontol. B Psychol. Sci. Soc. Sci.* 76 1937–1947. 10.1093/geronb/gbab014 33460446 PMC8598990

[B11] Flood M. (2002). Successful aging: a concept analysis. *J. Theory Constr. Test.* 6 105–108. 10.1177/0898010112463492 23072825

[B12] Frankl V. E. (1984). *Man’s Search for Meaning: an Introduction to Logotherapy (3rd ed.).* New York, NY: Simon & Schuster.

[B13] Gellis Z. McClive-Reed K. Kenaley B. Kim E. (2020). Meaning of life and well-being: preliminary results of the successful aging study. *Innov. Aging* 4:112. 10.1093/geroni/igaa057.369

[B14] Hayes A. F. (2017). *Introduction to Mediation, Moderation, and Conditional Process Analysis: a Regression-Based Approach.* New York, NY: Guilford Publications.

[B15] Holmbeck G. N. (2002). Post-hoc probing of significant moderational and mediational effects in studies of pediatric populations. *J. Pediatr. Psychol.* 27 87–96. 10.1093/jpepsy/27.1.87 11726683

[B16] Huang Y. (2020). Special issue: challenges of population ageing in China. *China Econ. J.* 13 1–2. 10.1080/17538963.2019.1710058

[B17] Hupkens S. Machielse A. Goumans M. Derkx P. (2018). Meaning in life of older persons. *Nurs. Ethics* 25 973–991. 10.1177/0969733016680122 30871429

[B18] Hyun Cha N. Ju Seo E. Sok S. R. (2012). Factors influencing the successful aging of older Korean adults. *Contemp. Nurse* 41 78–87. 10.5172/conu.2012.41.1.78 22724909

[B19] Jang E. S. Kim K. (2021). The mediating role of interpersonal needs on attitude towards ageing and its relationship with community sense and depression among community-dwelling older adults. *Health Soc. Care Commun.* 29 547–553. 10.1111/hsc.13117 32748424

[B20] Jin Y. He M. Li J. (2016). The relationship between meaning in life and subjective well-being in China: a meta-analysis. *Adv. Psychol. Sci.* 24 1854–1863. 10.3724/SP.J.1042.2016.01854

[B21] Keyes C. L. M. (2002). The Mental Health Continuum: from languishing to flourishing in life. *J. Health Soc. Behav.* 43 207–222. 10.2307/3090197 12096700

[B22] Kim S. H. Park S. (2016). A meta-analysis of the correlates of successful aging in older adults. *Res. Aging* 39 657–677. 10.1177/0164027516656040 27334287

[B23] Kinory D. Aisenberg D. Levi-Belz Y. (2020). The cost of being apart. *J. Nerv. Ment. Dis.* 208 663–670. 10.1097/nmd.0000000000001198 32520851

[B24] Kwon Y. S. Kim S. C. Lee Y. R. Hyun M. H. (2020). Effects of thwarted interpersonal needs and alcohol consumption on physical pain tolerance. *Soc. Behav. Pers.* 48 1–13. 10.2224/sbp.8563

[B25] Kyron M. J. Hooke G. R. Bryan C. J. Page A. C. (2021). Distress tolerance as a moderator of the dynamic associations between interpersonal needs and suicidal thoughts. *Suicide Life Threat. Behav* [Epub online ahead of print] 10.1111/sltb.12814 34741322

[B26] Lai C. C. W. Boag S. (2021). Chinese versions of the interpersonal needs questionnaire: psychometric properties, measurement invariance across gender and cultures. *PsyCh. J.* 10 635–648. 10.1002/pchj.436 33655693

[B27] Lee J. E. Kahana E. Kahana B. Zarit S. (2022). The role of goal and meaning in life for older adults facing interpersonal stress. *Aging Ment. Health* 26 149–158. 10.1080/13607863.2020.1849020 33939563

[B28] Liu S. S. Gan Y. Q. (2010). Reliability and validity of the Chinese version of the meaning in life questionnaire. *Chin. Ment. Health J.* 24 478–482. 10.3969/j.issn.1000-6729.2010.06.021

[B29] Luo H. Ren X. Li J. Wu K. Li N. (2020). Association between obesity status and successful aging among older people in China: evidence from CHARLS. *BMC Public Health* 20:767. 10.1186/s12889-020-08899-9 32448262 PMC7245862

[B30] Macià D. Cattaneo G. Solana J. Tormos J. M. Pascual-Leone A. Bartrés-Faz D. (2021). Meaning in life: a major predictive factor for loneliness comparable to health status and social connectedness. *Front. Psychol.* 12:627547. 10.3389/fpsyg.2021.627547 33716892 PMC7943478

[B31] Melton A. M. A. Schulenberg S. E. (2007). On the relationship between meaning in life and boredom proneness: examining a Logotherapy postulate. *Psychol. Rep.* 101 1016–1022. 10.2466/pr0.101.4.1016-1022 18361113

[B32] National Bureau of Statistics of China (2021). *Bulletin of the Seventh National Census (No. 5).* Available online at: http://www.stats.gov.cn/tjsj/tjgb/rkpcgb/qgrkpcgb/202106/t20210628_1818824.html (accessed on May 11, 2021)

[B33] Park Y. Kim H. S. (2019). The interaction between personality and interpersonal needs in predicting suicide ideation. *Psychiatry Res.* 272 290–295. 10.1016/j.psychres.2018.12.091 30594762

[B34] Podsakoff P. M. MacKenzie S. B. Lee J. Y. Podsakoff N. P. (2003). Common method biases in behavioral research: a critical review of the literature and recommended remedies. *J. Appl. Psychol.* 88 879–903. 10.1037/0021-9010.88.5.879 14516251

[B35] Rowe J. W. Kahn R. L. (2015). Successful aging 2.0: conceptual expansions for the 21st century. *J. Gerontol. B Psychol. Sci. Soc. Sci.* 70 593–596. 10.1093/geronb/gbv025 25878054

[B36] Shields M. (2008). Community belonging and self-perceived health. *Health Rep.* 19 51–60. 18642519

[B37] Shim Y. Choe K. Kim K. Kim J. Ha J. (2021). The applicability of the interpersonal-psychological theory of suicide among community-dwelling older persons. *Suicide Life Threat. Behav.* 51 816–823. 10.1111/sltb.12757 33870547

[B38] Shiovitz-Ezra S. Litwin H. (2012). Social network type and health-related behaviors: evidence from an American national survey. *Soc. Sci. Med.* 75 901–904. 10.1016/j.socscimed.2012.04.031 22682660 PMC3552155

[B39] Soares M. U. Facchini L. A. Nedel F. B. Wachs L. S. Kessler M. Thumé E. (2021). Social relationships and survival in the older adult cohort. *Rev. Lat. Am. Enfermagem* 29:e3395. 10.1590/1518-8345.3844.3395 33439948 PMC7798399

[B40] Steger M. F. Frazier P. Oishi S. Kaler M. (2006). The meaning in life questionnaire: assessing the presence of and search for meaning in life. *J. Couns. Psychol.* 53 80–93. 10.1037/0022-0167.53.1.80

[B41] Steger M. F. Kawabata Y. Shimai S. Otake K. (2008). The meaningful life in Japan and the United States: levels and correlates of meaning in life. *J. Res. Pers.* 42 660–678. 10.1016/j.jrp.2007.09.003

[B42] Steger M. F. Mann J. R. Michels P. Cooper T. C. (2009). Meaning in life, anxiety, depression, and general health among smoking cessation patients. *J. Psychosom. Res.* 67 353–358. 10.1016/j.jpsychores.2009.02.006 19773029

[B43] Stoeckel K. J. Litwin H. (2016). The impact of social networks on the relationship between functional impairment and depressive symptoms in older adults. *Int. Psychogeriatr.* 28 39–47. 10.1017/S1041610215000538 25943121

[B44] Troutman M. Nies M. A. Bentley M. (2011a). Measuring successful aging in southern black older adults. *Educ. Gerontol.* 37 38–50. 10.1080/03601277.2010.500587

[B45] Troutman M. Nies M. A. Small S. Bates A. (2011b). The development and testing of an instrument to measure successful aging. *Res. Gerontol. Nurs.* 4 221–232. 10.3928/19404921-20110106-02 21261228

[B46] Van Orden K. A. Cukrowicz K. C. Witte T. K. Joiner T. E. (2012). Thwarted belongingness and perceived burdensomeness: construct validity and psychometric properties of the Interpersonal Needs Questionnaire. *Psychol. Assess.* 24 197–215. 10.1037/a0025358 21928908 PMC3377972

[B47] Van Orden K. A. Witte T. K. Cukrowicz K. C. Braithwaite S. R. Selby E. A. Joiner T. E. (2010). The interpersonal theory of suicide. *Psychol. Rev.* 117 575–600. 10.1037/a0018697 20438238 PMC3130348

[B48] Wang M. C. Dai X. Y. (2008). Chinese Meaning in Life Questionnaire revised in college students and its reliability and validity test. *Chin. J. Clin. Psychol.* 16 459–461. 10.3969/j.issn.1673-4254.2017.04.13 28446403 PMC6744089

[B49] Wang R. Chen Y. Hu F. Wang Z. Cao B. Xu C. (2021). Psychometric Properties of Interpersonal Needs Questionnaire-15 for predicting suicidal ideation among migrant industrial workers in China. *Int. J. Env. Res. Pub. He.* 18:7583. 10.3390/ijerph18147583 34300033 PMC8306592

[B50] Wang Y. Zhou C. (2020). Promoting social engagement of the elderly to cope with aging of the Chinese population. *Biosci. Trends* 14 310–313. 10.5582/bst.2020.03305 32848106

[B51] Yang X. Fan C. Liu Q. Lian S. Cao M. Zhou Z. (2019). The mediating role of boredom proneness and the moderating role of meaning in life in the relationship between mindfulness and depressive symptoms. *Curr. Psychol.* 40 4635–4646. 10.1007/s12144-019-00408-5

[B52] Ye L. Zhang X. (2021). The association mechanism between social network types and health-related behaviours among the elderly in rural Hubei Province. *China. Int. J. Health Plann. Manag.* 36 826–846. 10.1002/hpm.3125 33598957

[B53] Yin K. L. He J. M. (2012). Reliability and validity of the Mental Health Continuum Short Form in adults. *Chin. Ment. Health J.* 26 388–392. 10.3969/j.issn.1000-6729.2012.05.015

[B54] Yu Y. Xiao Y. (2021). Coparenting alleviated the effect of psychological distress on parental psychological flexibility. *Front. Psychol.* 12:646380. 10.3389/fpsyg.2021.646380 34335362 PMC8322113

[B55] Yu Y. Yu Y. Hu J. (2021). COVID-19 among Chinese high school graduates: psychological distress, growth, meaning in life and resilience. *J. Health Psychol.* [Epub online ahead of print] 10.1177/1359105321990819 33541149 PMC8685742

[B56] Zhong M. Zhang Q. Bao J. Xu W. (2019). Relationships between meaning in life, dispositional mindfulness, perceived stress, and psychological symptoms among Chinese patients with gastrointestinal cancer. *J. Nerv. Ment. Dis.* 207 34–37. 10.1097/nmd.0000000000000922 30575706

[B57] Zhou Y. Xu W. (2019). Short report: the mediator effect of meaning in life in the relationship between self-acceptance and psychological wellbeing among gastrointestinal cancer patients. *Psychol. Health Med.* 24 725–731. 10.1080/13548506.2018.1554252 30514112

